# Effects of *Gastrodia Elata* Bl on Phencyclidine-Induced Schizophrenia-Like Psychosis in Mice

**DOI:** 10.2174/157015911795017263

**Published:** 2011-03

**Authors:** E.-J Shin, J.-M Kim, X.-K. T Nguyen, T.-T. L Nguyen, S. Y Lee, J.-H Jung, M. J Kim, W. K Whang, K Yamada, T Nabeshima, H.-C Kim

**Affiliations:** 1 Neuropsychopharmacology and Toxicology Program, College of Pharmacy, Kangwon National University, Chunchon 200-701, South Korea; 2 Oriental Bio-Herb Research Institute, Kangwon National University, Chunchon 200-701, South Korea; 3 Phamaceutical Resources Botany, College of Pharmacy, Chung-Ang University, Seoul 156-756, South Korea; 4 Department of Neuropsychopharmacology and Hospital Pharmacy, Nagoya University Graduate School of Medicine, Nagoya 466-8560, Japan; 5 Department of Chemical Pharmacology, Graduate School of Pharmaceutical Sciences, Meijo University, Nagoya 468-8503, Japan

**Keywords:** *Gastrodia elata* Bl, phencyclidine, schizophrenia, 5-HT_1A_ receptors.

## Abstract

It has been demonstrated that 5-HT_1A_ receptors play an important role in the pathophysiology of schizophrenia. Because *Gastrodia elata *Bl (GE) modulates the serotonergic system, we examined whether GE could affect phencyclidine (PCP)-induced abnormal behavior in mice. Repeated treatment with PCP increased immobility time, while it decreased social interaction time and recognition memory. PCP-induced abnormal behaviors were significantly attenuated by GE, and these effects were comparable to those of 8-OH-DPAT, a 5-HT_1A_ receptor agonist. Furthermore, GE-mediated effects were counteracted by WAY 100635, a 5-HT_1A_ receptor antagonist. Our results suggest that the antipsychotic effects of GE are, at least in part, mediated *via* activation of 5-HT_1A_ in mice.

## INTRODUCTION

Schizophrenia is a chronic, devastating, and costly mental illness, affecting about 1% of the world population. It develops progressively, and is often undetected during childhood and adolescence in a premorbid phase, leading to the onset of psychosis in early adulthood [1].

Phencyclidine [1-(1-phenylcyclohexyl)piperidine hydrochloride (PCP)], a non-competitive N-methyl-D-aspartate (NMDA) antagonist, has been shown to induce schizophrenia-like psychosis, with positive symptoms, negative symptoms, and cognitive deficits in humans [2], which persist for several weeks after withdrawal from chronic PCP use [3].

To understand the pathophysiology of schizophrenia, an animal model of schizophrenia was established using PCP [3]. Nabeshima and colleagues previously demonstrated that repeated treatment with PCP induces several behavioral abnormalities, such as increased immobility in a forced swimming test, social deficits on a social interaction test, impairment of latent learning in a water finding test, and associative learning impairment in cue and contextual fear conditional tests in mice [3]. Thus, PCP-treated mice might be a useful animal model of schizophrenia.

Several lines of evidence have suggested that serotonin 5-HT_1A_ receptors may play a role in the pathophysiology of psychiatric diseases, including schizophrenia, and that 5-HT_1A_ receptors might be an important target for emotion and cognition [4].

*Gastrodia elata* Blume (GE) is a well-known herbal agent that has long been used to treat headache, paralysis, migraine, and other neurological disorders in traditional oriental medicine. Major components of GE include gastrodin, p-hydroxybenzyl aldehyde, p-hydroxybenzylalcohol, vanillyl alcohol, and vanillin. Earlier reports indicated that GE has various biological properties, including anti-convulsant, anti-oxidant, cognitive enhancing, anxiolytic, and anti-depressant effects [5]. Recently, it was demonstrated that GE significantly decreased immobility duration in a forced-swimming test in rats, primarily by modulating the serotonergic system [6].

Thus, to extend the pharmacological investigation of GE, we examined whether GE affected PCP-induced changes in immobility, social interaction, and cognitive function in mice. We also examined whether the 5-HT_1A_ receptor is involved in GE-mediated pharmacological actions in response to PCP.

## METHODS

All animals were treated in accordance with the NIH *Guide for the care and use of laboratory animals* (NIH Publication No. 85-23, 1985; www.dels.nas.edu/ila). This study was performed in accordance with the Institute for Laboratory Animal Research (ILAR) guidelines for the care and use of laboratory animals.

Male C57BL/6J mice or male ICR mice (Bio Genomic Inc., Charles River Technology, Gapyung-Gun, Gyeonggi-Do, South Korea), weighing 25±3 g, were maintained on a 12:12 h light:dark cycle and fed *ad libitum*. Male ICR mice were only used as the “target” in the social interaction test, with no drug treatment.

N-[2-[4-(2-methoxyphenyl)-1-piperazinyl]ethyl-N-(2-pyridinyl)cyclohexane carboxamide trihydrochloride (WAY 100635; Sigma-Aldrich, St. Louis, MO, USA), PCP hydrochloride (Tocris Bioscience, Ellisville, MO, USA), and (+)-8-hydroxy-2-(di-n-propylamino)tetralin (8-OH-DPAT; Sigma-Aldrich) were dissolved in 0.9% sterile saline. The GE was obtained from Samsung Herb Medicine, Co. (Chunchon, South Korea) and was suspended in 0.5% carboxymethylcellulose. All solutions were prepared immediately before use. Experimental schedules are shown in Fig. (**1**).

The novel object recognition, forced swimming, and social interaction tests were performed as described previously [6,7,8]. An automated video-tracking system (Noldus Information Technology, Wageningen, The Netherlands) was used to record and analyze the movements of mice in all three tests.

Statistical analyses were performed using one-way analysis of variance (ANOVA) or repeated measure one-way ANOVA. A *post-hoc* Fisher’s PLSD test was then applied. A *P* value < 0.05 was deemed to indicate statistical significance.

## RESULTS AND DISCUSSION

**Fig. (2):**
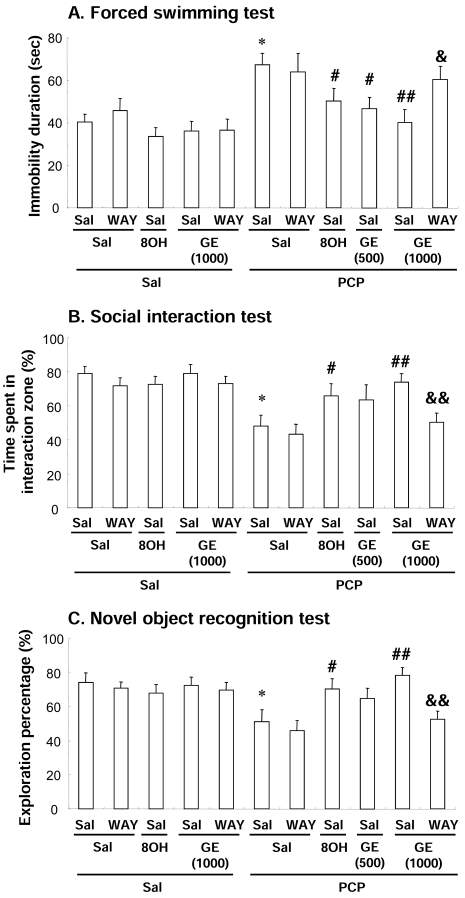
Effect of WAY 100635 (WAY) on the GE-mediated pharmacological actions in response to PCP-induced changes in the immobility time (A), social interaction time (B), and recognition memory (C). Sal = saline, GE (500) = GE 500 mg/kg, p.o., GE (1000) = GE 1000 mg/kg, p.o., 8OH = 8-OH-DPAT 0.5 mg/kg. i.p., WAY = WAY 100635 0.5 mg/kg, i.p. Each value is the mean ± S.E.M. of 12 mice. ^*^ *P* < 0.01 vs. Saline + Saline + Saline, ^#^ *P* < 0.05, ^##^ *P* < 0.01 vs. Saline + Saline + PCP, ^&^ *P* < 0.05, ^&&^ *P* < 0.01 vs. Saline + GE (1000) + PCP (One-way ANOVA followed by Fisher’s PLSD test).

Repeated treatment with PCP resulted in significant increases (*P* < 0.01) in immobility time in the forced swimming test (Fig. **2A**), while PCP resulted in significant decreases (*P* < 0.01) in the interaction time in the social interaction test (Fig. **2B**) and exploration rate for a novel object (*P* < 0.01) in the novel object recognition test (Fig. **2C**). GE treatment significantly attenuated PCP-induced increase in immobility time [GE (500 mg/kg) + PCP vs. saline + PCP, *P* < 0.05; GE (1000 mg/kg) + PCP vs. saline + PCP, *P* < 0.01] (Fig. **2A**), sociability deficit [GE (1000 mg/kg) + PCP vs. saline + PCP, *P* < 0.01] (Fig. **2B**), and impaired visual recognition memory [GE (1000 mg/kg) + PCP vs. saline + PCP, *P* < 0.01] (Fig. **2C**), in a dose-dependent manner. The effects of GE (8-OH-DPAT + PCP vs. saline + PCP, *P* < 0.05 in all behaviors) were comparable to those of 8-OH-DPAT (0.05 mg/kg, i.p.), a 5-HT_1A_ receptor agonist. Consistently, WAY 100635 (0.5 mg/kg, i.p.), a 5-HT_1A_ receptor antagonist, significantly inhibited GE-mediated pharmacological actions [forced swimming test: WAY 100635 + GE (1000 mg/kg) + PCP vs. saline + GE (1000 mg/kg) + PCP, *P* < 0.05; social interaction test and novel object recognition test: WAY 100635 + GE (1000 mg/kg) + PCP vs. saline + GE (1000 mg/kg) + PCP, *P* < 0.01] in response to PCP (Fig. **2**). These results suggest that GE attenuated PCP-induced changes in immobility time, social interaction, and cognitive function *via* modulation of 5-HT_1A_ receptors.

Our results are consistent with earlier findings that repeated treatment with PCP showed significant increases in immobility time and significant decreases in social interaction and recognition memory in mice [3]. Prolonged exposure to GE significantly blocked PCP-induced behavioral effects, in a dose-related manner. The protective effects of GE in response to PCP were about equipotent to those of the 5-HT_1A_ receptor agonist 8-OH-DPAT (0.05 mg/kg, i.p.). Furthermore, the 5-HT_1A_ receptor antagonist WAY 100635 (WAY; 0.5 mg/kg, i.p.) significantly counteracted GE-mediated pharmacological effects in response to PCP. Thus, we believe that GE-mediated activation of 5-HT_1A_ receptors may be important in antipsychotic effects in response to PCP, although this remains to be explored further in other GE-mediated neuropharmacological activities.

Recent finding have suggested that repeated PCP treatment significantly decreases the density of 5-HT_1A_ receptors in the mouse brain [9]. 5-HT_1A_ agonist properties are thought to improve negative symptoms and cognitive deficits by stimulating the release of dopamine in the prefrontal cortex [10]. Consistent with this, Sumiyoshi *et al.* [11] reported that the 5-HT_1A_ agonists exert anti-depressant-like effects [12], of particular interest for schizophrenic patients suffering from depression. Recent reports have indicated that potential antipsychotic effects on PCP require combined modulation of 5-HT_1A_ and dopamine receptors [8]. Interestingly, the GE-mediated anti-depressant effects are exerted, at least in part, by dopaminergic modulation in the rat brain [6]; however, the interaction between 5-HT_1A_ and specific dopamine receptors remains to be determined.

Similar to GE, various 5HT_1A_ receptor agonists, such as buspirone, 8-OH-DPAT, and ipsapirione, have been shown to enhance social interaction [13]. Furthermore, various preclinical data strengthen the notion that targeting the 5HT_1A_ receptor system should result in beneficial effects on dysfunctional social behavior, possibly not only in schizophrenic patients but also in the population suffering from social withdrawal of other etiologies.

Hagiwara *et al.* [9] demonstrated that the hippocampal density of 5-HT1A receptor is much higher than the frontal cortical density of 5-HT receptor in mice, and that repeated treatment with PCP did not significantly alter the frontal cortical density of 5-HT, but did change the hippocampal density of 5-HT receptors, and that perospirone, a 5-HT_1A_ receptor agonist, ameliorated PCP-induced cognitive deficits, as measured by a novel object recognition test. Thus, the cognitive enhancing effect of GE or 8-OH-DPAT may be similar to that of perospirone. It remains to be determined whether GE also modulates hippocampal 5-HT_1A_ receptors in our experimental system.

Atypical antipsychotic drugs, such as clozapine, zipraidone, aripiprazole, and quetiapine, are all 5-HT_1A_ receptor (partial) agonists, which may be relevant for their actions in treating schizophrenia [14]. While current antipsychotic treatments are effective against positive symptoms, they have significant side effects and have little effect on negative or cognitive symptoms [15].

In conclusion, our finding suggests that 5-HT_1A_ receptor agonistic properties of GE offer potential therapeutic advantages in response to PCP-induced schizophrenia-like psychosis, although many details of the GE-mediated effect(s) remain to be determined.

## Figures and Tables

**Fig. (1):**
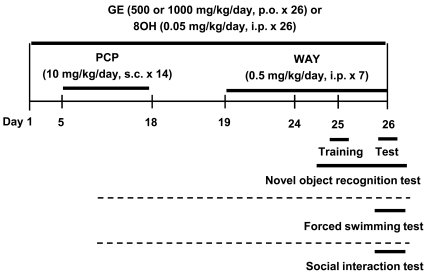
Experimental schedules. PCP = phencyclidine, GE = *Gastrodia elata* Blume, 8OH = 8-OH-DPAT, WAY = WAY 100635. 8-OHDPAT was used as a reference drug. Mice were treated with PCP (10 mg/kg/day, s.c.) for 14 consecutive days. After a 7- or 8-day withdrawal period, the novel object recognition, forced swimming, and social interaction tests were performed using independent sets of mice; each set of mice was used for one of the three behavioral tests. Treatment with GE (500 or 1000 mg/kg/day, p.o.) or 8-OH-DPAT (0.05 mg/kg/day, i.p.) was started from 4 days before the first PCP injection, and continued throughout the experimental period. WAY 100635 (0.5 mg/kg/day, i.p.) was administered during the PCP withdrawal period. GE was injected 1 h prior to PCP or WAY 100635, and WAY 100635 was injected 30 min prior to the behavior test.
